# Supplementary material for: Chromothripsis during telomere crisis is independent of NHEJ, and consistent with a replicative origin
Source: Genome Res. 2019 May;29(5):737–49. doi: 10.1101/gr.240705.118 (PMC6499312; doi:10.1101/gr.240705.118)
Supplement: Supplemental Material [file supp_gr.240705.118_Supplemental_file_1.zip › contigs/annotated_contigs/DB71/contig.3.DB71_length_575_mean_cov_4.95130434783.docx]

**DB71_length_575_mean_cov_4.95130434783**

```
AGGATACAAACAAATGGAAGAACATTCCATGCTCATGGGTAGGAAGAATCAATATCGTGAAAATGGCCTTAATGCCCAAGGTAATTTAT
 >chrX:57174563-57174858 - E=8e-154
AGATTCAATGCCATCCCCATCAAGCTACCAATGACTTTCTTCATAGAATTGGAAAAAACTACTTTAAAGTTCATATGGAACCAAAAAGG

AGCCTGCATCACCAAGTTAAACCTAAGCCAAAAAAACAAAGCCGGAGGCATCATGCTACCTGACTTCAAACTATACTACAAGGCTACAG

TAACCAAAACAGCATGGTACTGGTACC|A|TATATATATATATGATCATATATATAT|G|ATCACCAATGATCAGGGAAATGTTAATCA
                            >chr12:65500126-65500154 - E=4e-05 p=8e-03   >chrX:571714
AAACCACAATGTAATACCACCTTACCCCCCCCAAAAGAATGATCATAATCAAAAAACAAAAACTAATAGATGCTGGTATGGATGTGGTG
95-57171748 - E=9e-136
AAAAGGGAACACTTCTACACTGCTGGTGGGAATGTAAACTAGTACAACCACTAGAAAAAACAGTGTGGAGATTCCTTAGGGAACTAAAA

GTAGAACTAACATTTGATCCAGCAATCCCACTACCCACAGAAAAA
```
